# The new era of treatments for kidney transplant recipients

**DOI:** 10.1590/2175-8239-JBN-2026-0018en

**Published:** 2026-08-17

**Authors:** Helio Tedesco Silva

**Affiliations:** 1 Universidade Federal de São Paulo, Escola Paulista de Medicina, Divisão de Nefrologia, São Paulo, SP, Brazil.; 2 CTI Clinical Trial and Consulting Services, São Paulo, SP, Brazil.

**Keywords:** Kidney Transplantation, Immunosuppression Therapy, Biological Products, Complement Inhibitors, Signaling Lymphocytic Activation Molecule Receptors.

## Abstract

Despite substantial improvements in short-term outcomes after kidney transplantation, important unmet clinical needs persist. These include expanding transplant access for highly sensitized candidates, mitigating the short- and long-term consequences of ischemia–reperfusion injury, preventing acute rejection while preserving allograft function, minimizing off-target toxicities associated with calcineurin inhibitor–based immunosuppressive regimens, improving adherence to long-term immunosuppressive therapy, and developing effective treatments for late antibody-mediated rejection. A growing pipeline of novel therapeutic agents aims to address these challenges by targeting key immunologic and injury pathways that contribute to allograft dysfunction and failure. In this review, we examine emerging pharmacologic strategies currently undergoing clinical evaluation in these domains and critically assess their potential to improve long-term graft survival and patient outcomes. We also discuss the key considerations and priorities that should inform future clinical investigation in kidney transplantation.

## Key Points

**Unmet medical needs in kidney transplantation**

- Increasing access to transplantation for highly sensitized patients.
- Development of selective lymphocyte-depleting induction therapies that favor a tolerogenic immunological environment following homeostatic reconstitution.
- Promotion of faster and more complete recovery of kidney function in the immediate post-transplant period.
- Prevention of acute rejection through CNI-free strategies, enabling stable kidney function, improved adherence, and avoidance of CNI-associated systemic and nephrotoxic long-term adverse effects.
- Effective treatment of late acute rejection, reducing the risk of graft loss and prolonging graft survival.

**Novel therapies**

- Protease-induced transient IgG cleavage, targeted depleting agents, complement inhibitors, costimulatory blockers, and selective B- and NK-cell inhibitors are under development.

**Implications**

- The impact on clinical practice is expected to be substantial, particularly with the chronic use of biologic agents administered either in clinic-based settings or through home self-administration, alongside anticipated improvements in patient and graft survival.

**Future perspectives**

- The incorporation of these therapies will depend on real-world confirmation of the efficacy and safety observed in clinical trials, regional cost-effectiveness analyses, identification of optimal strategies (including combined or sequential approaches), characterization of patient subgroups most likely to benefit, and integration of patient-reported outcomes and quality of life.

## CURRENT MEDICAL NEED

Kidney transplantation is associated with improved survival compared with remaining on dialysis among patients with advanced chronic kidney disease. However, long-term survival among kidney transplant recipients remains lower than that of age-, sex-, and comorbidity-matched individuals in the general population^1,2^. Excess mortality after transplantation is largely attributable to complications related to immunosuppression, including infection, malignancy, and cardiovascular disease, as well as the cumulative effects of pre-existing chronic kidney disease^3,4^.

Several areas continue to be associated with suboptimal outcomes in kidney transplantation. These include transplantation in highly sensitized recipients, delayed graft function, prevention of acute rejection, and treatment of late antibody-mediated rejection. Ongoing research and drug development efforts are directed toward these clinical challenges.

## METHOD

This review was not designed to provide an exhaustive summary of all investigational therapies in kidney transplantation. Drugs and biological agents were selected according to the following criteria: (1) relevance to the clinical needs outlined above; and (2) evidence of an established clinical development program with multicenter trials registered in ClinicalTrials.gov and a plausible pathway toward regulatory approval.

Small single-center studies and early proof-of-concept investigations were not included. The characteristics of the drugs and biological agents discussed in this review are summarized in Table 1.

**Table 1.** Characteristics of drug and biological products under development

Drug/biological	Biological function	Indication	Development	Primary endpoint	Safety concerns	ClinicalTrials.gov identifier
Imlifidase (recombinant cysteine protease enzyme)	Elimination of IgG antibodies, including DSAs	Highly sensitized patients (cPRA >90%) – Europe	Conditional approval	Access to transplantation	Allergic reactions, DSA rebound and rejection, increased risk of serious infections, and thromboembolic events	N/A
		cPRA ≥99.9% – USA	Phase 3	Mean estimated glomerular filtration rate (eGFR) at 12 months		NCT04935177
Ravulizumab	C5 inhibitor	DGF	Phase 3 (enrolling)	Time to freedom from dialysis through 90 days post-transplant	Bacterial infection; Mandatory vaccination/antibiotic prophylaxis.	NCT06830798
Empasiprubart	C2 inhibitor	DGF	Phase 2 (enrollment completed)	eGFR at 24 weeks post-transplant	Bacterial infection; Mandatory vaccination/antibiotic prophylaxis.	NCT05907096
Pegcetacoplan	C3 inhibitor	DGF	Phase 3	Time to freedom from dialysis through 90 days post-transplant	Bacterial infection; Mandatory vaccination/antibiotic prophylaxis.	NCT07020832
Siplizumab	Anti-CD2	Induction therapy	Phase 2	Incidence of adverse events (AEs), adverse events of special interest (AESIs), and infections	Viral infection (EBV)	NCT04311632
Tegoprubart	Anti-CD40L	Prophylaxis of acute rejection	Phase 2 (completed enrollment)	Regulatory composite endpoint	Viral infection (EBV, CMV, and BKV); Thromboembolic events.	NCT05983770
Frexalimab	Anti-CD40L	Prophylaxis of acute rejection	Phase 2/3	Regulatory composite endpoint	Viral infection (EBV, CMV, and BKV); Thromboembolic events.	NCT07412470
TNX-1500	Anti-CD40L	Prophylaxis of acute rejection	Phase 2	Incidence of all AEs and serious adverse events (SAEs)	Viral infection (EBV, CMV, and BKV); Thromboembolic events.	NCT07204080
Pegrizeprument	Anti-CD28	Prophylaxis of acute rejection	Phase 1 completed	Incidence of SAEs; Incidence of treatment-emergent adverse events (TEAEs).	Viral infection (EBV, CMV, and BKV) Infusion-related reactions, infections, and hypogammaglobulinemia	NCT07290777
Felzartamab	Anti-CD38	Treatment of AMR/cAMR	Phase 3	Biopsy-proven histologic resolution	Infusion-related reactions, infections, and hypogammaglobulinemia	NCT06685757/NCT07219043
ALXN203	C3 inhibitor	Treatment of AMR/cAMR	Phase 2	Biopsy-proven histologic resolution	Bacterial infection; Mandatory vaccination/antibiotic prophylaxis.	NCT06744647

## INCREASING ACCESS TO KIDNEY TRANSPLANTATION FOR HIGHLY SENSITIZED PATIENTS

Highly sensitized patients have a higher risk of antibody-mediated rejection and graft loss following kidney transplantation. However, with appropriate allocation and desensitization strategies, transplantation in this population can result in outcomes superior to those associated with continued dialysis^2,5,6^.

Imlifidase is a recombinant cysteine protease derived from *Streptococcus pyogenes* that cleaves human immunoglobulin G (IgG) antibodies at the hinge region. This enzymatic activity results in rapid and near-complete degradation of circulating IgG, eliminating Fc-mediated effector functions within hours after intravenous administration. This mechanism permits transplantation in patients with preformed donor-specific antibodies^7^.

The drug is administered as a single intravenous dose, typically 0.25 mg/kg, before transplantation to achieve rapid IgG clearance and enable HLA-incompatible transplantation. The pharmacologic effect is transient, with recovery of IgG levels occurring within 1-2 weeks as endogenous antibody synthesis resumes. Clinical considerations include the potential for rebound of donor-specific antibodies after transplantation and the need to coordinate the timing of induction immunosuppression, as imlifidase can also cleave therapeutic IgG antibodies^8^.

Imlifidase has received conditional approval in Europe for use in highly sensitized patients (calculated panel reactive antibody [cPRA] ≥90%) with limited likelihood of receiving an HLA-compatible transplant^9,10^. An open-label, controlled, randomized phase 3 trial has just been completed (NCT04935177). The study evaluated 12-month kidney function in highly sensitized kidney transplant recipients (cPRA ≥99.9%) with a positive crossmatch against a deceased donor and compared desensitization with imlifidase versus standard of care. The treatment cost is substantial, with a list price of approximately £300,490 per course. Nevertheless, its economic value should be assessed within a comprehensive cost-effectiveness framework. Key considerations include the relatively small population of highly sensitized patients eligible for treatment, as well as potential downstream cost offsets arising from reduced time on the transplant waiting list, lower comorbidity burden, and decreased waiting-list mortality. Importantly, the expected prolongation of dialysis-free survival is likely to translate into significant gains in quality-adjusted life years (QALYs). These factors should be incorporated into incremental cost-effectiveness ratio (ICER) analyses to determine whether the intervention represents good value for money relative to the current standard of care.

## STRATEGIES TO PROMOTE RECOVERY OF KIDNEY FUNCTION

### Delayed graft function and clinical outcomes

Delayed graft function (DGF) is associated with adverse long-term outcomes after kidney transplantation, including reduced graft function, increased risk of acute rejection, graft loss, and mortality^11^. Large cohort studies and meta-analyses indicate that DGF is associated with an approximately 41% increased risk of graft loss within three years after transplantation and with higher rates of acute rejection and lower long-term kidney function^2,12,13^.

Prolonged DGF, defined as dialysis requirement for more than 14 days, is associated with substantially lower graft and patient survival compared with shorter DGF episodes or immediate graft function^14,15,16^. Incomplete recovery from DGF, characterized by reduced glomerular filtration rate (GFR) at one month after transplantation, independently predicts an increased risk of graft loss^17^.

Risk factors for DGF involve donor, recipient, and perioperative variables^18,19^.

**Donor-related factors** include deceased donor status, particularly kidneys from donation after circulatory death (DCD), advanced donor age, higher body mass index, hemodynamic instability, use of vasoactive agents, reduced urine output, and elevated terminal serum creatinine.

**Recipient-related factors** include longer duration of dialysis, diabetes mellitus, male sex, obesity, African American race, human leukocyte antigen mismatching, and preformed anti-HLA antibodies.

**Perioperative factors** include the distance between organ procurement and the transplant center and prolonged cold ischemia time.

Several of these factors are modifiable, including optimization of donor hemodynamic stability and reduction of cold ischemia time^20^.

### The role of machine perfusion

In deceased donor kidney transplantation, hypothermic machine perfusion (HMP) is associated with a lower incidence of delayed graft function compared with static cold storage (SCS). Reported relative risk reductions range from 20% to 40%, corresponding to a number needed to treat of approximately 8–13, depending on donor characteristics and cold ischemia time^21,22^.

These benefits have been observed in kidneys recovered after brain death and after circulatory death, across varying cold ischemia times, and in more recent transplant cohorts^21,22^.

Reduced rates of DGF with machine perfusion are associated with improved short- and long-term graft survival. Economic analyses suggest that machine perfusion is cost-effective, particularly for higher-risk grafts such as kidneys from expanded-criteria donors or those exposed to prolonged cold ischemia^21,23^.

The timing and duration of perfusion influence its effectiveness. Continuous perfusion from procurement to implantation appears to provide greater benefit than brief periods of end-ischemic perfusion^24^. Recent analyses indicate that improvements in graft survival associated with machine perfusion are largely mediated through reductions in DGF.

### Role of the complement system in ischemia–reperfusion injury

Complement activation contributes to delayed graft function (DGF) after kidney transplantation through its role in ischemia–reperfusion injury (IRI). During transplantation, hypoxia followed by reperfusion activates complement locally within renal tubular cells, primarily through the lectin and alternative pathways. Collectin-11, a renal lectin, recognizes glycan ligands expressed on hypoxic cells and initiates complement activation through the lectin pathway, with subsequent amplification via the alternative pathway, resulting in inflammation and tissue injury^25,26,27^.

Formation of the terminal complement complex (C5b-9) is increased perioperatively in patients who develop DGF, with levels reported to be two to three times higher than in recipients with early or slow graft function. Elevated C5b-9 concentrations are associated with adverse short- and long-term graft outcomes^28,29^. Complement activation products, including C3a, C5a, and C5b-9, promote local inflammation, recruit immune cells, and contribute to tubular epithelial cell dysfunction, thereby amplifying graft injury^26,30^.

### Targeting the complement system to improve recovery of kidney function

C1 esterase inhibitor (C1-INH) is a plasma glycoprotein of the serine protease inhibitor (serpin) family that regulates multiple proteolytic cascades, including the complement pathways, the contact (kallikrein–kinin) system, the intrinsic coagulation pathway, and fibrinolysis. C1-INH inactivates proteases such as C1r, C1s, factor XIIa, plasma kallikrein, and factor XIa, thereby limiting activation of these pathways and the generation of mediators such as bradykinin^31^.

In a phase I/II double-blind placebo-controlled trial, kidney transplant recipients at risk for DGF (n = 35 per group) received C1-INH at a dose of 50 units/kg, before reperfusion of the allograft, and again 24 hours later (maximum 4000 units per dose). Treatment was not associated with a statistically significant reduction in the incidence of DGF compared with placebo (44.1% vs. 60.0%; P = 0.232). Laboratory parameters including C3, C4, fibrinogen, prothrombin time, partial thromboplastin time, and D-dimer levels did not differ between groups. However, C1-INH treatment was associated with a shorter duration of DGF and a higher estimated glomerular filtration rate (eGFR) at 12 months (57.33 ± 15.77 vs. 44.90 ± 20.52 ml/min per 1.73 m^2^; P = 0.006)^32^. At 3.5 years of follow-up, mean kidney function remained higher in the C1-INH group (56 vs. 35 ml/min per 1.73 m^2^). Graft failure occurred more frequently in the placebo group (n = 7) than in the C1-INH group (n = 1), whereas deaths were fewer in the placebo group (1 vs. 3)^33^.

Eculizumab is a humanized monoclonal antibody that inhibits terminal complement activation by binding complement component C5. This interaction prevents the formation of the membrane attack complex (C5b-9) and the generation of the anaphylatoxin C5a, both of which contribute to inflammatory and cytotoxic responses during ischemia–reperfusion injury. Inhibition of terminal complement activation reduces complement-mediated inflammation, endothelial injury, and thrombosis associated with transplanted organs^27,34^.

In the phase 2/3 PROTECT trial (ClinicalTrials.gov identifier NCT02145182), 288 kidney transplant recipients at increased risk for DGF were randomized at 77 centers to receive eculizumab (n = 142; 1200 mg immediately before reperfusion and 900 mg within 18–24 hours) or placebo (n = 143). No significant differences were observed between groups in the composite outcome of DGF, graft loss, or loss to follow-up (35.9% vs. 41.7%; P = 0.38). The incidence of DGF (33.8% vs. 39.6%), eGFR at 1 month (42.6 ± 20.8 vs. 40.0 ± 20.3 ml/min per 1.73 m^2^), and graft survival (94.9% vs. 91.0%) were also similar between the eculizumab and placebo groups. Experimental data suggest that incomplete suppression of C5 activation may limit the pharmacodynamic effect of eculizumab in this setting^35^.

Ravulizumab is a modified monoclonal antibody derived from eculizumab that differs by four amino acids, resulting in increased affinity for the neonatal Fc receptor and an extended half-life. Ravulizumab provides sustained inhibition of complement component C5 with less frequent dosing compared with eculizumab while maintaining comparable efficacy and safety in approved indications^36^. These pharmacokinetic characteristics have led to the initiation of a phase 3 trial evaluating ravulizumab in kidney transplant recipients at high risk for DGF (the AWAKE trial). The primary endpoint is time to freedom from dialysis through 90 days after transplantation (ClinicalTrials.gov identifier NCT06830798).

Empasiprubart is a humanized recycling monoclonal antibody that binds the CCP2 domain of complement factor 2 (C2), reducing circulating C2 levels by up to 99%. The elimination half-life ranges from 70 to 88 days, resulting in prolonged dose-dependent inhibition of the classical and lectin complement pathways^37^. A phase 2 multicenter trial evaluating the safety, efficacy, and tolerability of empasiprubart in recipients of deceased donor kidney transplants at risk for DGF has completed recruitment. The primary endpoint is the eGFR at 24 weeks after transplantation (ClinicalTrials.gov identifier NCT05907096).

Pegcetacoplan is a pegylated cyclic peptide that binds complement component C3 and its activation fragment C3b, preventing C3 cleavage and downstream complement activation. This proximal inhibition blocks activation of all three complement pathways by preventing formation of C3 and C5 convertases, thereby reducing complement-mediated inflammation and tissue injury. The standard dosing regimen consists of 1080 mg administered by subcutaneous infusion twice weekly^38^. A phase 3 clinical trial evaluating pegcetacoplan in adults at high risk of DGF following kidney transplantation is expected to enroll approximately 320 participants. The primary endpoint is time to freedom from dialysis through 90 days after transplantation (ClinicalTrials.gov identifier NCT07020832).

### Critical considerations

The complement system is a central component of innate immunity and contributes to host defense, inflammation, and tissue repair. Complement inhibition in the setting of kidney transplantation raises several clinical and mechanistic considerations.

#### Timing of complement inhibition

Complement activation may occur before transplantation. Brain death and circulatory death in organ donors activate complement pathways and may initiate ischemic injury before organ recovery. Most clinical studies administer complement inhibitors before graft reperfusion. Alternative approaches, including *ex vivo* perfusion of the graft with complement inhibitors or treatment of donors before organ procurement, require further investigation.

#### Selection of complement targets

The optimal complement component or pathway for therapeutic inhibition remains uncertain. Preclinical studies have not established whether inhibition of the classical, lectin, or alternative pathways provides the most effective strategy for preventing ischemia–reperfusion injury while maintaining safety. The degree of complement inhibition required for clinical efficacy is also unclear. Residual C5 activity and persistent upstream complement activation may contribute to the limited efficacy observed with terminal pathway inhibition in some studies.

#### Patient selection

Clinical trials have focused on kidney transplant recipients at increased risk for DGF, although inclusion and exclusion criteria vary among studies. The potential interaction between complement inhibition and other interventions that reduce DGF risk, such as hypothermic or normothermic machine perfusion, has not been fully evaluated.

#### Outcome measures

Primary endpoints differ across clinical trials and include the incidence of DGF, duration of dialysis dependence, and kidney function at 90 to 180 days after transplantation. This heterogeneity limits direct comparison across studies.

#### Safety

Systemic complement inhibition is associated with increased susceptibility to encapsulated bacterial infections. Vaccination and prophylactic antibiotic strategies are commonly recommended during periods of complement inhibition, particularly in transplant recipients receiving concurrent immunosuppressive therapy.

#### Regulatory considerations

The development of multiple complement inhibitors targeting different components of the pathway and the use of heterogeneous clinical endpoints present challenges for regulatory evaluation. The clinical significance of treatment effects, particularly for short-term outcomes such as DGF, will need to be balanced against potential safety risks.

#### Cost-effectiveness

Short-term economic benefits may arise from reduced dialysis requirements during episodes of DGF. Long-term cost-effectiveness will depend on sustained improvements in graft function, reductions in graft failure, and the overall safety profile of complement inhibition strategies.

#### Future perspectives

Considering the initial heterogeneous results observed with complement inhibitors in improving kidney function recovery after transplantation, uncertainties regarding the optimal timing of administration, the level of pathway inhibition, appropriate outcome measures, and their interplay with current and emerging machine perfusion strategies suggest that the ideal clinical indication, if any, will need to await the results of phase 3 clinical trials.

## PREVENTION OF ACUTE REJECTION

### Limitations of calcineurin inhibitor–based immunosuppressive regimens

Tacrolimus in combination with mycophenolate, with or without corticosteroids, remains the standard maintenance immunosuppressive regimen in kidney transplantation^39^. Induction therapy with interleukin-2 receptor (IL-2R) antagonists or anti-thymocyte globulin is commonly used based on the estimated risk of acute rejection and delayed graft function^40^. These strategies have resulted in high one-year patient and graft survival rates.

Despite these favorable short-term outcomes, several limitations of calcineurin inhibitor (CNI)–based regimens have been identified. High intra-individual variability in tacrolimus trough concentrations and reduced time within the therapeutic range are associated with an increased risk of graft loss, irrespective of medication adherence^41,42^. In addition, CNIs are associated with nephrotoxicity and metabolic and cardiovascular adverse effects that may compromise long-term graft outcomes^43^.

Anti-thymocyte globulin has recently received regulatory approval for prophylaxis of acute rejection in kidney transplant recipients^44^. However, its use is associated with a dose-dependent increase in adverse events, including bacterial and viral infections^45^.

Long-acting biologic agents may provide more stable pharmacodynamic effects and simplified dosing regimens. Such characteristics may improve treatment adherence and potentially contribute to improved long-term graft outcomes^46^.

### Anti-CD2 therapy

Siplizumab is a humanized monoclonal antibody targeting CD2 that has been investigated in induction therapy and tolerance protocols. Its mechanism of action includes selective depletion of effector memory T cells, reduced T-cell activation and proliferation, and relative preservation or enrichment of regulatory T cells (Tregs), which may promote immune tolerance and reduce alloimmune responses^47,48^.

Phase 2 trials evaluating the safety, tolerability, pharma­cokinetics, and pharmacodynamics of escalating doses of siplizumab compared with rabbit anti-thymocyte globulin in *de novo* kidney transplant recipients have been completed (NCT06365437; NCT04311632). Phase 2b/3 clinical trials are anticipated.

### Costimulatory blockade

Effective alloimmune responses require costimulatory signals generated by interactions between antigen-presenting cells and T lymphocytes^49^. Costimulatory pathways involve receptors within the immunoglobulin superfamily (CD80, CD86, CD28, CTLA-4, ICOS, ICOSL, PD-1, and PD-L1) and the tumor necrosis factor superfamily (CD40 and CD154)^50^.

Biologic agents targeting costimulatory pathways selectively interfere with membrane-bound immune receptors and may reduce non-immune off-target effects compared with broader immunosuppressive strategies.

### CD40–CD40L pathway

CD40 and CD40 ligand (CD40L, CD154) are expressed on multiple cell types. The receptor CD40 is constitutively expressed on B cells and myeloid cells, whereas CD40L is primarily expressed on activated T cells and platelets. Interaction between CD40 and CD40L activates signaling pathways that regulate immune responses, inflammation, hematopoiesis, thrombosis, and tumor cell biology^51^.

Inhibition of CD40–CD40L signaling interferes with both cellular and humoral alloimmune responses. The pathway is required for full T-cell activation and for B-cell activation and differentiation, including the generation of donor-specific antibodies. Blockade of this interaction using monoclonal antibodies directed against CD154 or CD40 prolongs allograft survival and reduces rejection in preclinical transplantation models^52^.

Anti-CD154 antibodies exert several immunologic effects, including inhibition of CD40–CD154 signaling, reduction of B-cell clonal expansion and antibody production, modulation of innate immune cell activation, and promotion of inducible regulatory T cells^52^. Preclinical studies suggest that CD154 blockade may be more effective than CD40 blockade in preventing rejection. A meta-analysis in non-human primate transplantation models reported approximately threefold higher tolerance induction with anti-CD154 therapy compared with anti-CD40 agents^50^.

Clinical development of two anti-CD40 antibodies, iscalimab and ASKP1240, was discontinued due to insufficient clinical benefit.

### Anti-CD154 therapies

Early anti-CD154 antibodies were associated with an increased risk of thromboembolic events. This complication is thought to result from CD154 expression on activated platelets and the interaction between the antibody Fc region and platelet FcγRIIa receptors, leading to platelet activation and aggregation. Newer agents have been engineered with modified Fc domains or as Fab fragments to reduce platelet activation and mitigate thromboembolic risk^52^.

Tegoprubart is a second-generation humanized IgG1 antibody targeting CD154 with a modified Fc region designed to minimize FcγRIIa interaction. It is currently being evaluated in phase 2 trials for the prevention of acute rejection after kidney transplantation (NCT05983770).

Frexalimab is another second-generation anti-CD154 humanized monoclonal antibody under investigation for autoimmune diseases and transplantation^53^.

TNX-1500 is a third-generation anti-CD154 antibody currently in early-phase clinical development for kidney transplantation (NCT07204080).

### CD80/CD86–CD28–CTLA-4 pathway

CD28 and cytotoxic T-lymphocyte-associated protein 4 (CTLA-4) regulate T-cell activation through interactions with CD80 and CD86 expressed on antigen-presenting cells.

This receptor is constitutively expressed on naïve T cells and provides a critical costimulatory signal required for T-cell activation, proliferation, cytokine production, and survival following engagement of the T-cell receptor. CD28 signaling promotes clonal expansion and metabolic activation of T cells^54^.

CTLA-4 is upregulated following T-cell activation and binds CD80 and CD86 with higher affinity than CD28. Its signaling inhibits T-cell activation and proliferation through both cell-intrinsic and cell-extrinsic mechanisms, including trans-endocytosis of CD80/CD86 from antigen-presenting cells and modulation of regulatory T-cell function^55^.

Belatacept, a fusion protein composed of the extracellular domain of CTLA-4 linked to the Fc fragment of IgG1, selectively blocks CD28-mediated costimulation. Although belatacept is approved for kidney transplantation, its broader use has been limited by a higher incidence of acute rejection compared with CNI-based regimens^56^.

### Anti-CD28 therapy

Selective CD28 blockade may provide immunomodulation while preserving inhibitory CTLA-4 signaling. Because CD28 blockade does not interfere with CTLA-4–mediated coinhibitory signaling or PD-L1–CD80 interactions, regulatory T-cell function may be preserved, potentially improving control of alloimmune responses^57,58,59^.

Pegrizeprument (FR104) is a pegylated monovalent humanized Fab′ antibody fragment that antagonizes CD28 signaling. By selectively blocking CD28-mediated T-cell activation while preserving CTLA-4 and PD-L1 pathways, it may maintain regulatory immune mechanisms^60^.

Early-phase clinical studies have evaluated intravenous doses ranging from 0.02 mg/kg to 1.5 mg/kg, demonstrating dose-dependent receptor occupancy and pharmacodynamic effects^61^.

A phase 1/2 clinical trial (NCT04837092) is evaluating the safety, tolerability, pharmacokinetics, and preliminary efficacy of pegrizeprument in kidney transplant recipients receiving standard-criteria donor kidneys. A phase 2 trial (NCT07290777) will compare pegrizeprument with tacrolimus for the prevention of acute rejection following kidney transplantation.

### Critical assessment

Several challenges remain in the clinical development and implementation of costimulatory blockade in kidney transplantation.

#### Limitations of costimulatory blockade strategies

Despite a strong mechanistic rationale, the clinical development and adoption of costimulation blockers have been limited. Factors include increased rates of acute rejection, infectious complications (particularly viral infections), risk of post-transplant lymphoproliferative disorder (PTLD), uncertainties regarding long-term safety, and limited comparative efficacy relative to CNI-based regimens^49^.

#### Efficacy and safety of anti-CD154 antibodies

The greater efficacy of anti-CD154 therapies compared with anti-CD40 agents observed in non-human primate models require confirmation in clinical studies. Continued monitoring for thromboembolic events remains necessary despite Fc-engineering modifications in newer antibodies.

#### Efficacy and safety of anti-CD28 antibodies

The clinical efficacy of selective CD28 blockade requires further validation. The increased incidence of acute rejection observed with belatacept has been associated with circulating CD28-negative T cells, which may not be affected by CD28 antagonists^62^. In patients with belatacept-resistant rejection, the addition of an mTOR inhibitor has been associated with resolution of rejection and reduction of circulating CD8^+^CD28^−^ effector memory T cells^63^.

#### Therapeutic monitoring

Improved understanding of the pharmacokinetic and pharmacodynamic properties of biologic agents is required. Assessment beyond plasma drug concentrations and peripheral receptor occupancy may be necessary to optimize therapeutic strategies^56^.

#### Safety of costimulatory blockers

Safety considerations remain critical. These include risks of PTLD—particularly in Epstein–Barr virus–seronegative recipients treated with belatacept—and tuberculosis in regions with high endemic prevalence.

#### Challenges to demonstrating clinical superiority

Clinical trial design presents challenges. Demonstrating superiority using current regulatory composite endpoints (acute rejection, graft loss, death, or loss to follow-up) is difficult because of low event rates in contemporary control groups. Differences in kidney function at 12 months may also require large study populations to detect meaningful effects.

Although the clinical relevance of this composite endpoint has been debated^64^, the identification and validation of surrogate markers for long-term graft survival remain challenging. The *iBox* scoring system integrates clinical, functional, immunologic, and histologic parameters, including time from transplantation, estimated glomerular filtration rate, proteinuria, biopsy findings, and donor-specific antibodies^65^.

The *iBox* score measured at one year after transplantation demonstrated superior prognostic performance for graft loss compared with biopsy-proven acute rejection occurring within the first post-transplant year^66^. The *iBox* scoring system was qualified by the European Medicines Agency in 2022 as a secondary efficacy endpoint for kidney transplant clinical trials. Its qualification as a surrogate endpoint for regulatory decision-making is currently under review by the U.S. Food and Drug Administration. The use of the *iBox* score at 12 months as a co-primary endpoint representing a surrogate for long-term death-censored graft survival is under investigation.

#### Optimal immunosuppressive regimen

The optimal background immunosuppressive regimen for costimulatory blockade remains uncertain. Comparable rejection rates with standard therapy have been reported primarily with belatacept combined with an mTOR inhibitor^67,68^. Preclinical and translational studies suggest that the combination of costimulatory blockade and mTOR inhibition may promote donor-specific tolerance, prolonged graft survival, and stable mixed chimerism^69,70,71^.

However, the tolerability profile of mTOR inhibitors and regulatory considerations have limited further development of this strategy. In addition, cost-effectiveness analyses will be required to determine whether potential improvements in graft function and survival offset the costs associated with biologic therapies and long-term monitoring.

## TREATMENT OF ANTIBODY-MEDIATED REJECTION

Antibody-mediated rejection (AMR) is characterized by donor-specific antibody (DSA) binding to graft endothelial antigens, complement activation, endothelial injury, and recruitment of inflammatory effector cells, including natural killer (NK) cells. These processes result in microvascular inflammation and progressive allograft injury^72^.

A recent systematic review reported an incidence of acute AMR ranging from 1.1% to 21.5%, while chronic AMR occurs in approximately 7.5%–20.1% of recipients within 10 years after transplantation. AMR is an independent risk factor for death-censored graft failure^73^.

Current treatment recommendations are largely based on expert consensus. The Transplantation Society working group recommends plasmapheresis combined with intravenous immunoglobulin (IVIG) for early AMR, together with optimization of baseline immunosuppressive therapy. For late active or chronic active AMR, optimization of maintenance immunosuppression is generally recommended^74^.

Multiple phase II and III clinical trials are evaluating therapeutic strategies targeting different components of the humoral immune response, including inhibition of interleukin-6 (IL-6) signaling, depletion of B cells and plasma cells, enzymatic cleavage of circulating DSAs, selective complement inhibition, and CD38-targeted therapies^75,76^.

### Anti-CD38 therapies

CD38 is a multifunctional transmembrane glycoprotein expressed on a wide range of hematopoietic and non-hematopoietic cells. Expression increases following immune cell activation and is observed on T cells, B cells, and NK cells, where CD38 contributes to the regulation of cellular differentiation, proliferation, cytokine secretion, apoptosis, phagocytosis, chemotaxis, and NK-cell cytotoxic activity^77^.

CD38 also regulates intracellular nicotinamide adenine dinucleotide (NAD^+^) metabolism and influences mitochondrial and metabolic function^78^.

Targeting CD38 leads to depletion of plasma cells, plasmablasts, B cells, and NK cells. The mechanisms involved include antibody-dependent cellular cytotoxicity (ADCC), complement-dependent cytotoxicity (CDC), antibody-dependent cellular phagocytosis (ADCP), and inhibition of CD38 enzymatic activity. The relative contribution of these mechanisms depends on the binding epitope and the structure of the monoclonal antibody used^79^.

Felzartamab, daratumumab, and isatuximab are anti-CD38 monoclonal antibodies that have been used off-label for the treatment of AMR^75^.

A recent phase 2 double-blind, placebo-controlled study reported resolution of histologic AMR activity in 82% of patients treated with felzartamab at week 24 compared with 20% in the placebo group. Treatment was associated with reductions in histologic AMR activity, microvascular inflammation scores, interferon-γ–inducible transcripts, NK-cell gene signatures, and donor-derived cell-free DNA. No significant changes in DSA levels were observed, and only minimal effects on endothelial injury transcripts were detected. Molecular recurrence of rejection was observed approximately six months after treatment discontinuation^80^.

A phase 3 multicenter clinical trial evaluating felzartamab in kidney transplant recipients with late AMR is currently enrolling participants (NCT06685757).

### Complement inhibition and anti-C3 strategies

Complement activation is a key component of AMR pathogenesis. Binding of DSAs—most commonly IgG—to endothelial antigens activates the classical complement pathway, generating complement split products such as C4d and C3d. These fragments covalently bind to endothelial surfaces and serve as diagnostic markers of complement activation in AMR.

Formation of the membrane attack complex (C5b-9) can directly injure endothelial cells. However, recent data suggest that terminal complement activation may be less prominent in late or chronic AMR, whereas upstream complement fragments such as C4d and C3d are more consistently detected and may have greater clinical relevance^81^.

Evidence supporting the use of the C5 inhibitor eculizumab for the treatment of AMR remains limited, and routine use is not currently recommended. Alternative strategies targeting proximal complement components, including C1 esterase inhibitors and monoclonal antibodies such as sutimlimab, are under investigation.

RNA interference–based therapeutics represent an additional approach to complement inhibition. Small interfering RNA (siRNA) molecules induce post-transcriptional gene silencing by promoting degradation of target messenger RNA (mRNA). These double-stranded RNA molecules, typically 21–23 nucleotides in length, are incorporated into the RNA-induced silencing complex (RISC), where the antisense strand directs sequence-specific cleavage of complementary mRNA, thereby preventing translation and reducing protein expression^82^.

Several siRNA-based therapies have been approved for clinical use, including treatments for hereditary transthyretin amyloidosis (patisiran), acute hepatic porphyria (givosiran), primary hyperoxaluria type 1 (lumasiran), and familial hypercholesterolemia (inclisiran).

ALXN203 is a double-stranded non-coding siRNA conjugated to N-acetylgalactosamine (GalNAc) to enable hepatocyte-targeted delivery via the asialoglycoprotein receptor. The molecule reduces hepatic production of complement component C3 through degradation of C3 mRNA. A phase 2 multicenter clinical trial is currently recruiting kidney transplant recipients with AMR to evaluate the efficacy and safety of ALXN203 (NCT06744647).

### Critical assessment

Several factors complicate therapeutic development for AMR.

#### AMR heterogeneity

AMR is an uncommon, complex, and heterogeneous condition with evolving diagnostic criteria incorporating clinical, histologic, and molecular features. Microvascular inflammation with or without detectable DSAs has been associated with adverse graft outcomes^83^.

#### AMR activity

Histologic and molecular analyses demonstrate considerable variability in disease activity even when strict diagnostic criteria are applied. This variability may reflect distinct phenotypes or different stages of disease progression^84,85^.

#### Clinical trial challenges

This heterogeneity complicates clinical trial design and regulatory evaluation of novel therapies.

#### Chronic treatment

Emerging data suggest that sustained therapy may be required to maintain disease control, as recurrence of rejection has been observed after treatment discontinuation in early clinical studies^80^.

#### Long-term safety

The long-term safety profile of these therapies under chronic administration remains uncertain. Monitoring strategies, vaccination programs, antimicrobial prophylaxis, and surveillance for endemic infections will be necessary to evaluate the long-term risk–benefit balance and potential effects on quality of life.

## FINAL CONSIDERATIONS

**Figure 1:**
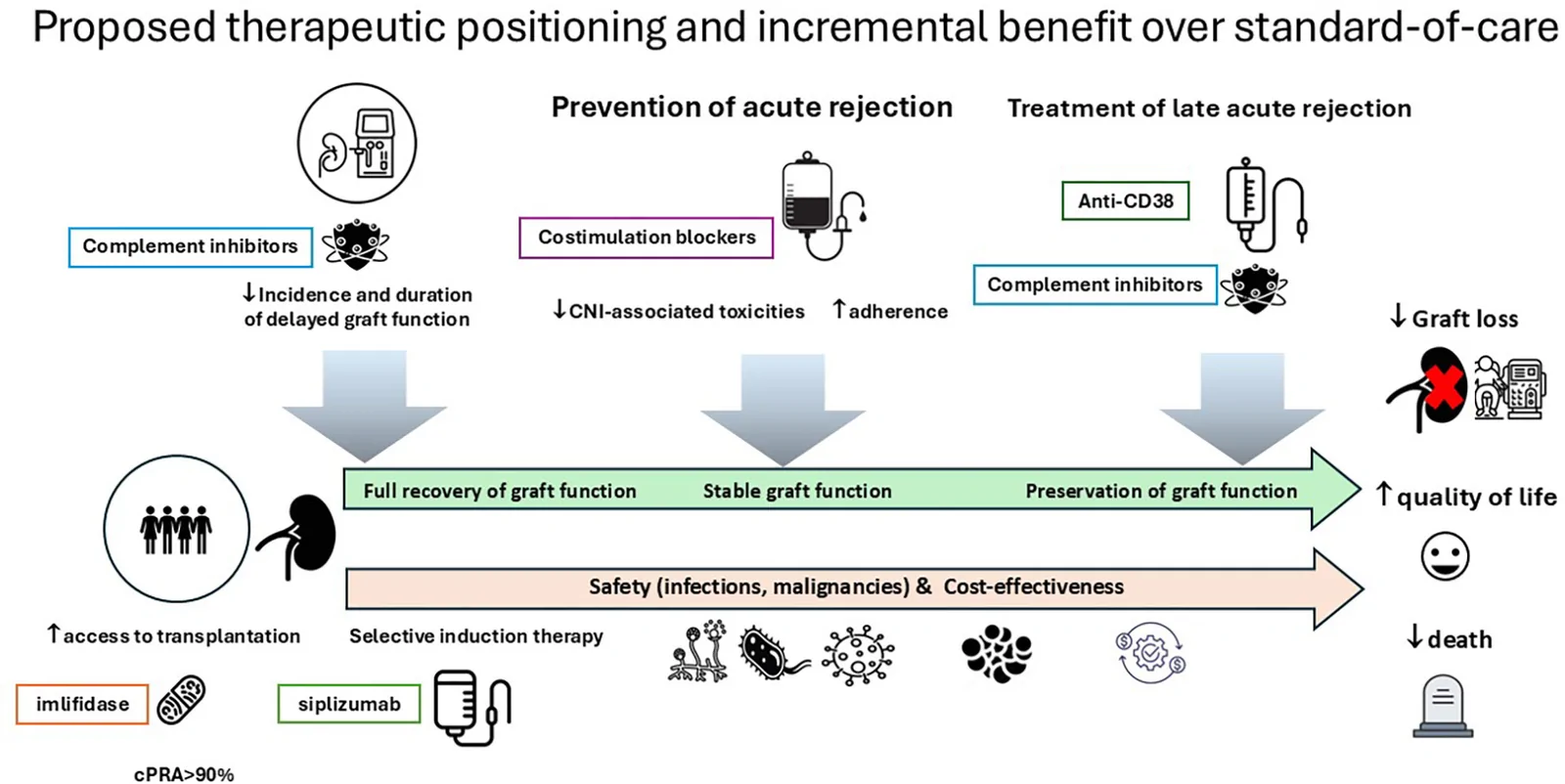
Envisioned therapeutic positioning of emerging agents in kidney transplantation.

The development of targeted immunotherapies represents an important area of investigation in kidney transplantation. Current therapeutic strategies under study address several major clinical challenges, including management of highly sensitized patients, selective pro-tolerogenic induction therapy, mitigation of ischemia–reperfusion injury, prevention of acute rejection without calcineurin inhibitor–related toxicities, and treatment of antibody-mediated rejection (Figure 1).

Emerging therapeutic strategies aim to provide incremental benefits over the current standard-of-care regimen, which consists of induction with rabbit antithymocyte globulin (rATG) and maintenance immunosuppression with tacrolimus, mycophenolate mofetil, and corticosteroids. Several approaches are under investigation. First, desensitization with imlifidase may expand access to transplantation for highly sensitized candidates. Second, inhibition of the complement cascade may reduce the incidence and duration of delayed graft function and promote more complete recovery of allograft function. Third, selective induction therapy with siplizumab may provide effective immunosuppression while reducing adverse events associated with rATG. Fourth, maintenance immunosuppression with novel costimulation pathway blockers may eliminate calcineurin inhibitor–associated metabolic, cardiovascular, and nephrotoxic toxicities while maintaining stable kidney function. In addition, costimulation blockade has been associated with improved survival of regulatory T (Treg) cells, reduced effector T-cell proliferation, diminished memory B-cell responses, and lower levels of donor-specific antibodies. Finally, emerging therapeutic approaches for the treatment of late acute and chronic active antibody-mediated rejection may slow the progression of kidney allograft dysfunction. These potential benefits must be balanced against the risks associated with intensified or novel immunosuppression, including bacterial, viral, and fungal infections, as well as oncogenic virus–associated malignancies such as post-transplant lymphoproliferative disorder. Rigorous benefit–risk and cost-effectiveness analyses will therefore be essential to determine the clinical value of these emerging strategies, with the goal of reducing graft loss, return to dialysis, and mortality, while improving the quality of life of kidney transplant recipients.

The challenges in translating the inhibition of promising mechanisms observed in preclinical studies into clinical benefits are well known in the transplant field. Early termination of development of fingolimod^86^, the JAK3 inhibitor tofacitinib^87^, and the tyrosine kinase inhibitor sotrastaurin^88^ are representative examples.

The future clinical role of these therapies remains uncertain, and their integration into standard immunosuppressive regimens will depend on the demonstration of efficacy, safety, and cost-effectiveness. Combination strategies targeting complementary immune pathways may be required to achieve optimal control of alloimmune responses.

Potential safety considerations include increased sus­ceptibility to bacterial infections associated with complement inhibition and viral infections associated with costimulatory blockade, as well as possible viral reactivation and oncogenic viral complications.

Long-term safety data are available for the C5 inhibitor eculizumab. Analysis of 46,316 adverse drug events reported in the U.S. Food and Drug Administration Adverse Event Reporting System identified 461 preferred terms consistent with product labeling, including fatigue, nasopharyngitis, meningococcal infection, fever, and anemia^89^.

Costimulatory blockade is associated with preservation of regulatory T cells, reduced effector T-cell proliferation, decreased memory B-cell responses, and lower levels of donor-specific antibodies. Because these therapies primarily inhibit naïve T-cell activation while sparing established memory responses, the incidence of opportunistic infections may depend on prior pathogen exposure and concomitant immunosuppressive therapy^90^.

Pharmacodynamic monitoring strategies for targeted biologic therapies remain limited. Current assessments are largely restricted to measurement of circulating complement components or receptor occupancy, which may not fully capture downstream immunologic effects.

The prolonged half-life and potential for subcutaneous administration of several emerging biologic agents may allow less frequent dosing and improved treatment adherence. Continued investigation will be required to determine optimal therapeutic combinations, sequencing strategies, and long-term safety profiles.

## Data Availability

No new data were generated or analyzed in this study.
