# Supplementary material for: The new era of treatments for kidney transplant recipients
Source: J Bras Nefrol. 2026 Aug 17;48(4):e20260018. doi: 10.1590/2175-8239-JBN-2026-0018en (PMC13491757; doi:10.1590/2175-8239-JBN-2026-0018en)
Supplement: Translation

# Translation: A nova era dos tratamentos para receptores de transplante renal

**DOI:** 10.1590/2175-8239-JBN-2026-0018pt

**Authors:** Helio Tedesco Silva

**Affiliations:** 1 Universidade Federal de São Paulo, Escola Paulista de Medicina, Divisão de Nefrologia, São Paulo, SP, Brasil.; 2 CTI Clinical Trial and Consulting Services, São Paulo, SP, Brasil.

**Keywords:** Transplante de Rim, Terapia de Imunossupressão, Produtos Biológicos, Inibidor de Complemento, Moléculas de Sinalização da Ativação Linfocitária.

## Abstract

Apesar das substanciais melhorias nos resultados clínicos de curto prazo após o transplante renal, diversos desfechos clínicos intermediários ainda apresentam resultados insatisfatórios e necessitam de terapias médicas direcionadas. Estes incluem ampliar o acesso ao transplante para candidatos altamente sensibilizados, mitigar as consequências de curto e longo prazo da lesão de isquemia–reperfusão, prevenir a rejeição aguda, preservando simultaneamente a função do aloenxerto, minimizar as toxicidades associadas aos regimes imunossupressores baseados em inibidores da calcineurina, melhorar a adesão à terapia imunossupressora de longo prazo e desenvolver tratamentos eficazes para a rejeição tardia mediada por anticorpos. Um número crescente de novos agentes terapêuticos em desenvolvimento busca abordar esses desafios, interferindo direta e seletivamente em vias imunológicas e de lesão que contribuem para a disfunção e a falência do aloenxerto. Nesta revisão, examinamos estratégias farmacológicas emergentes atualmente em avaliação clínica e avaliamos criticamente seu potencial para melhorar a sobrevida do enxerto a longo prazo e os desfechos dos pacientes. Também discutimos considerações e prioridades que devem orientar futuras investigações clínicas no campo do transplante renal.

## Pontos-chave

**Necessidades médicas não atendidas em transplante renal**

1. Acesso ao transplante para pacientes altamente sensibilizados.
2. Desenvolvimento de terapias de depleção de linfócitos seletivas que promovem um ambiente imunológico tolerogênico após a reconstituição homeostática.
3. Promoção da recuperação mais rápida e completa da função renal no período imediatamente após o transplante.
4. Prevenção da rejeição aguda por meio de estratégias sem inibidores da calcineurina, possibilitando a estabilidade da função renal, a melhora da adesão e a eliminação dos efeitos adversos sistêmicos e nefrotóxicos associados ao uso crônico dos inibidores da calcineurina.
5. Tratamento eficaz da rejeição aguda tardia, reduzindo a perda progressiva de função renal e prolongando a sobrevida do enxerto.

**Terapias inovadoras**

1. Clivagem transitória de IgG induzida por proteases, agentes depletores seletivos, inibidores do complemento, bloqueadores de coestimulação e inibidores seletivos de células B e NK estão em desenvolvimento.

**Implicações**

2. Espera-se que o impacto na prática clínica seja substancial, especialmente com o uso crônico de agentes biológicos admini­strados tanto em clínicas quanto por autoadministração domiciliar, além do aumento da sobrevida do paciente e do enxerto.

**Perspectivas futuras**

3. A incorporação dessas terapias dependerá da confirmação clínica em mundo real da eficácia e segurança observadas em ensaios clínicos, de análises regionais de custo-efetividade, da identificação de estratégias combinadas ou sequenciais, da caracterização dos subgrupos de pacientes com maior benefício e da integração dos resultados relatados pelos pacientes, incluindo dados de qualidade de vida.

## NECESSIDADE MÉDICA ATUAL

O transplante renal está associado a uma melhora na sobrevida quando comparado à permanência em diálise entre pacientes com doença renal crônica avançada. No entanto, a sobrevida de longo prazo entre receptores de transplante renal permanece inferior à observada na população geral, pareada por idade, sexo e comorbidades^1,2^. O excesso de mortalidade após o transplante é amplamente atribuível a complicações relacionadas à imunossupressão, incluindo infecções, neoplasias e doenças cardiovasculares, bem como aos efeitos clínicos deletérios e cumulativos da doença renal crônica pré-existente^3,4^.

Diversas condições clínicas continuam associadas a desfechos subótimos no transplante renal. Entre elas, destacam-se o transplante em receptores altamente sensibilizados, a função tardia do enxerto, a prevenção da rejeição aguda e o tratamento da rejeição aguda tardia mediada por anticorpos. Esforços contínuos de pesquisa e desenvolvimento de medicamentos estão direcionados para reduzir o impacto desses desfechos clínicos na sobrevida do receptor de transplante renal.

## MÉTODO

Esta revisão não foi concebida para fornecer um resumo abrangente de todas as terapias em investigação no transplante renal. Fármacos e agentes biológicos foram selecionados de acordo com os seguintes critérios:

(1) relevância para as necessidades clínicas descritas acima; e (2) evidência de um programa de desenvolvimento clínico estabelecido, com estudos multicêntricos registrados no ClinicalTrials.gov e uma via plausível para aprovação regulatória.

Pequenos estudos de centro único e investigações iniciais de prova de conceito não foram incluídos. As características dos fármacos e agentes biológicos discutidos nesta revisão estão resumidas na Tabela 1.

**Tabela 1:** Características dos medicamentos e produtos biológicos em desenvolvimento

Droga/biológico	Função biológica	Indicação	Desenvolvimento	Desfecho primário	Segurança	Identificador do ClinicalTrials.gov
Imlifidase (enzima protease cisteína recombinante)	Eliminação de anticorpos IgG, incluindo DSAs	Pacientes altamente sensibilizados (cPRA >90%) –Europa	Aprovação condicional	Acesso ao transplante	Reação alérgica, rebote e rejeição mediada por DSA, aumento do risco de infecções graves e eventos tromboembólicos.	N/A
		Pacientes altamente sensibilizados (cPRA >99,9%) –Estados Unidos	Fase 3	TFGe aos 12 meses após o transplante		NCT04935177
Ravulizumabe	Inibidor de C5	DGF	Fase 3 (inclusão)	Tempo para independência de diálise até 90 dias após o transplante	Infecção bacteriana; Vacinação obrigatória/profilaxia antibiótica.	NCT06830798
Empasiprubarte	Inibidor de C2	DGF	Fase 2 (inclusão concluída)	TFGe às 24 semanas após o transplante	Infecção bacteriana; Vacinação obrigatória/profilaxia antibiótica.	NCT05907096
Pegcetacoplan	Inibidor de C3	DGF	Fase 3	Tempo para a independência de diálise até 90 dias após o transplante	Infecção bacteriana; Vacinação obrigatória/profilaxia antibiótica.	NCT07020832
Siplizumabe	Anti-CD2	Terapia de indução	Fase 2	Incidência de eventos adversos (EAs), eventos adversos de interesse especial (AESIs) e infecções	Infecção viral (EBV)	NCT04311632
Tegoprubarte	Anti-CD40L	Profilaxia da rejeição aguda	Fase 2 (inclusão concluída)	Desfecho composto regulatório	Infecção viral (EBV, CMV, BKV); Eventos tromboembólicos.	NCT05983770
Frexalimabe	Anti-CD40L	Profilaxia da rejeição aguda	Fase 2/3	Desfecho composto regulatório	Infecção viral (EBV, CMV, BKV); Eventos tromboembólicos.	NCT07412470
TNX-1500	Anti-CD40L	Profilaxia da rejeição aguda	Fase 2	Incidência de EAs e eventos adversos graves (SAEs)	Infecção viral (EBV, CMV, BKV); Eventos tromboembólicos.	NCT07204080
Pegrizeprumente	Anti-CD28	Profilaxia da rejeição aguda	Fase 1 concluída	Incidência de SAEs e incidência de eventos adversos emergentes ao tratamento (TEAEs)	Infecção viral (EBV, CMV, BKV)	NCT07290777
Felzartamabe	Anti-CD38	Tratamento da RMA/cRMA	Fase 3	Resolução histológica comprovada por biópsia	Reações relacionadas à infusão, infecções e hipogamaglobulinemia	NCT06685757/NCT07219043
ALXN203	Inibidor de C3 hepático	Tratamento da RMA/cRMA	Fase 2	Resolução histológica comprovada por biópsia	Infecção bacteriana; Vacinação obrigatória/profilaxia antibiótica.	NCT06744647

## AUMENTO DO ACESSO AO TRANSPLANTE RENAL PARA PACIENTES ALTAMENTE SENSIBILIZADOS

Pacientes altamente sensibilizados apresentam maior risco de rejeição mediada por anticorpos e perda do enxerto após o transplante renal. No entanto, com estratégias adequadas de alocação e dessensibilização, o transplante nessa população pode resultar em desfechos superiores aos associados à manutenção em diálise^2,5,6^.

A imlifidase é uma cisteíno-protease recombinante derivada de *Streptococcus pyogenes* que cliva anticorpos humanos da imunoglobulina G (IgG). Essa atividade enzimática resulta em degradação rápida e quase completa da IgG circulante, eliminando as funções efetoras mediadas pela fração Fc poucas horas após a administração intravenosa. Esse mecanismo permite a realização de transplante em pacientes com anticorpos pré-formados específicos contra o doador^7^.

O fármaco é administrado como dose intravenosa única de 0,25 mg/kg, antes do transplante, para alcançar rápida depuração da IgG e permitir o transplante HLA incompatível. O efeito farmacológico é transitório, com recuperação dos níveis de IgG ocorrendo em 1 a 2 semanas, à medida que a síntese endógena de anticorpos é retomada. Considerações clínicas incluem o potencial de rebote de anticorpos específicos contra o doador após o transplante e a necessidade de coordenar o momento da imunossupressão de indução, uma vez que a imlifidase também pode clivar anticorpos terapêuticos do tipo IgG^8^.

A imlifidase recebeu aprovação condicional na Europa para uso em pacientes altamente sensibilizados (painel de anticorpos reativos calculado [cPRA] ≥90%) com baixa probabilidade de receber um transplante HLA compatível^9,10^. Um ensaio clínico de fase 3, aberto, controlado e randomizado, avaliando a função renal em 12 meses em pacientes altamente sensibilizados (cPRA ≥99,9%) submetidos a transplante renal com *crossmatch* positivo contra o rim de doador falecido, comparando a dessensibilização com imlifidase ao tratamento padrão, foi recentemente concluído (NCT04935177).

O custo do tratamento é substancial, aproximadamente £ 300.490. No entanto, seu valor econômico deve ser avaliado dentro de um arcabouço abrangente de custo-efetividade. Entre os principais aspectos a serem considerados estão a população relativamente pequena de pacientes altamente sensibilizados elegíveis para o tratamento, bem como os potenciais ganhos indiretos decorrentes da redução do tempo em lista de espera para transplante, como a redução de comorbidades e da diminuição da mortalidade em lista. Importante destacar que o prolongamento esperado da sobrevida livre de diálise provavelmente se traduzirá em ganhos significativos em anos de vida ajustados pela qualidade (*quality-adjusted life years* — QALYs). Esses fatores devem ser incorporados em análises da razão de custo-efetividade incremental (*incremental cost-effectiveness ratio* — ICER) para determinar se a intervenção representa uma boa relação custo-benefício em comparação com o tratamento padrão atual.

## ESTRATÉGIAS PARA PROMOVER A RECUPERAÇÃO DA FUNÇÃO RENAL

### Função tardia do enxerto e desfechos clínicos

A função tardia do enxerto (*delayed graft function* – DGF) está associada a desfechos adversos de longo prazo após o transplante renal, incluindo redução da função do enxerto e maior risco de rejeição aguda, perda do enxerto e morte^11^. Grandes estudos de coorte e meta-análises indicam que a DGF está associada a um aumento aproximado de 41% no risco de perda do enxerto dentro de três anos após o transplante, além de maiores taxas de rejeição aguda e menor função renal em longo prazo^2,12,13^.

A DGF prolongada, definida como necessidade de diálise por mais de 14 dias, está associada a sobrevida substancialmente menor do enxerto e do paciente quando comparada a episódios mais curtos de DGF ou à função imediata do enxerto^14,15,16^. A recuperação incompleta da DGF, caracterizada por taxa de filtração glomerular (TFG) reduzida um mês após o transplante, prediz de forma independente um maior risco de perda do enxerto^17^.

Os fatores de risco para DGF envolvem variáveis relacionadas ao doador, ao receptor e ao período perioperatório^18,19^.

Fatores relacionados ao doador incluem:

- doador falecido, especialmente rins provenientes de doação após morte circulatória (DCD);
- idade avançada do doador;
- maior índice de massa corporal;
- instabilidade hemodinâmica;
- uso de agentes vasoativos;
- redução do débito urinário;
- creatinina sérica final elevada.

Fatores relacionados ao receptor incluem:

- maior tempo em diálise;
- diabetes mellitus;
- sexo masculino;
- obesidade;
- raça afro-americana;
- incompatibilidade de antígenos leucocitários humanos (HLA);
- presença de anticorpos anti-HLA pré-formados.

Fatores perioperatórios incluem:

- distância entre o local de captação do órgão e o centro transplantador;
- tempo prolongado de isquemia fria.

Muitos desses fatores são potencialmente modificáveis, incluindo a otimização da estabilidade hemodinâmica do doador e a redução do tempo de isquemia fria^20^.

### O papel da perfusão em máquina

No transplante renal com doador falecido, a perfusão hipotérmica em máquina (*hypothermic machine perfusion* – HMP) está associada a menor incidência de DGF quando comparada ao armazenamento hipotérmico estático (*static cold storage* – SCS). Reduções relativas de risco relatadas variam de 20% a 40%, correspondendo a um número necessário para tratar de aproximadamente 8 a 13, dependendo das características do doador e do tempo de isquemia fria^21,22^.

Esses benefícios foram observados tanto em rins provenientes de doadores em morte encefálica quanto em morte circulatória, em diferentes tempos de isquemia fria e em coortes mais recentes de transplante^21,22^.

A redução das taxas de DGF com o uso de perfusão em máquina está associada à melhora da sobrevida do enxerto em curto e longo prazo. Análises econômicas sugerem que a perfusão em máquina é custo-efetiva, especialmente para enxertos de maior risco, como rins provenientes de doadores de critérios expandidos ou expostos a tempos prolongados de isquemia fria^21,23^.

O momento e a duração da perfusão influenciam sua eficácia. A perfusão contínua, desde a captação até o implante, parece proporcionar maior benefício do que períodos breves de perfusão após um período de isquemia fria variável^24^. Análises recentes indicam que as melhorias na sobrevida do enxerto associadas à perfusão em máquina são amplamente mediadas pela redução da DGF.

### Papel do sistema complemento na lesão de isquemia–reperfusão

A ativação do sistema complemento contribui para a DGF após o transplante renal por meio de seu papel na lesão de isquemia–reperfusão (*ischemia–reperfusion injury* – IRI). Durante o transplante, a hipóxia seguida de reperfusão ativa o sistema complemento localmente nas células tubulares renais, principalmente pelas vias da lectina e alternativa. A colectina-11, uma lectina renal, reconhece ligantes glicanos expressos em células hipóxicas e inicia a ativação do complemento pela via da lectina, com amplificação subsequente pela via alternativa, resultando em inflamação e lesão tecidual^25,26,27^.

A formação do complexo terminal do complemento (C5b-9) aumenta no período perioperatório em pacientes que desenvolvem DGF, com níveis relatados duas a três vezes maiores do que em receptores com função imediata ou lenta do enxerto. Concentrações elevadas de C5b-9 estão associadas a desfechos adversos do enxerto em curto e longo prazo^28,29^.

Produtos de ativação do complemento, incluindo C3a, C5a e C5b-9, promovem inflamação local, recrutam células imunes e contribuem para a disfunção das células epiteliais tubulares, amplificando a lesão do enxerto^26,30^.

### Inibição do sistema complemento como estratégia terapêutica para melhorar a recuperação da função renal

O inibidor da C1 esterase (C1-INH) é uma glicoproteína plasmática da família dos inibidores de serino-proteases (serpins) que regula múltiplas cascatas proteolíticas, incluindo as vias do complemento, o sistema de contato (calicreína–cinina), a via intrínseca da coagulação e a fibrinólise. O C1-INH inativa proteases como C1r, C1s, fator XIIa, calicreína plasmática e fator XIa, limitando a ativação dessas vias e a geração de mediadores como a bradicinina^31^.

Em um estudo de fase I/II duplo-cego controlado por placebo, receptores de transplante renal em risco para DGF (n = 35 por grupo) receberam C1-INH na dose de 50 unidades/kg antes da reperfusão do aloenxerto e novamente 24 horas depois (máximo de 4000 unidades por dose). O tratamento não reduziu significativamente a incidência de DGF em comparação ao placebo (44,1% vs. 60,0%; P = 0,232). Parâmetros laboratoriais, incluindo C3, C4, fibrinogênio, tempo de protrombina, tempo de tromboplastina parcial e D-dímero, não diferiram entre os grupos.

Entretanto, o tratamento com C1-INH esteve associado a menor duração da DGF e maior taxa de filtração glomerular estimada (TFGe) em 12 meses (57,33 ± 15,77 vs. 44,90 ± 20,52 ml/min/1,73 m^2^; P = 0,006)^32^. Após 3,5 anos de seguimento, a função renal média permaneceu maior no grupo tratado (56 vs. 35 ml/min/1,73 m^2^). A falência do enxerto ocorreu com maior frequência no grupo placebo (n = 7) em comparação com o grupo C1-INH (n = 1), enquanto o número de óbitos foi menor no grupo placebo (1 vs. 3)^33^.

O eculizumabe é um anticorpo monoclonal humanizado que inibe a ativação terminal do complemento ao se ligar ao componente C5. Essa interação impede a formação do complexo de ataque à membrana (C5b-9) e a geração da anafilatoxina C5a, ambos envolvidos nas respostas inflamatórias e citotóxicas durante a lesão de isquemia–reperfusão. A inibição da ativação terminal do complemento reduz inflamação mediada pelo complemento, lesão endotelial e trombose associadas a órgãos transplantados^27,34^.

No estudo PROTECT de fase 2/3 (ClinicalTrials.gov NCT02145182), 288 receptores de transplante renal com maior risco de DGF foram randomizados em 77 centros para receber eculizumabe (n = 142; 1200 mg imediatamente antes da reperfusão e 900 mg em 18–24 horas) ou placebo (n = 143). Não foram observadas diferenças significativas entre os grupos no desfecho composto de DGF, perda do enxerto ou perda de seguimento (35,9% vs. 41,7%; P = 0,38). A incidência de DGF (33,8% vs. 39,6%), a TFGe em 1 mês (42,6 ± 20,8 vs. 40,0 ± 20,3 ml/min/1,73 m^2^) e a sobrevida do enxerto (94,9% vs. 91,0%) também foram semelhantes entre os grupos. Dados experimentais sugerem que a supressão incompleta da ativação do C5 pode limitar o efeito farmacodinâmico do eculizumabe nesse contexto^35^.

O ravulizumabe é um anticorpo monoclonal modificado derivado do eculizumabe que difere por quatro aminoácidos, resultando em maior afinidade pelo receptor Fc neonatal e meia-vida prolongada. O ravulizumabe proporciona inibição sustentada do C5 com intervalos de dose menos frequentes, mantendo eficácia e segurança comparáveis nas indicações aprovadas^36^. Essas características farmacocinéticas levaram ao início de um estudo de fase 3 avaliando ravulizumabe em receptores de transplante renal com alto risco de DGF (estudo AWAKE). O desfecho primário é o tempo decorrido até a independência de diálise em até 90 dias após o transplante (ClinicalTrials.gov NCT06830798).

O empasiprubarte é um anticorpo monoclonal humanizado reciclável que se liga ao domínio CCP2 do fator 2 do complemento (C2), reduzindo os níveis circulantes de C2 em até 99%. A meia-vida de eliminação varia de 70 a 88 dias, resultando em inibição prolongada dependente da dose das vias clássica e da lectina do complemento^37^. Um estudo multicêntrico de fase 2 avaliando a segurança, a eficácia e a tolerabilidade do empasiprubarte em receptores de transplante renal de doador falecido em risco de DGF já concluiu o recrutamento. O desfecho primário é a taxa de filtração glomerular estimada (TFGe) em 24 semanas após o transplante (ClinicalTrials.gov NCT05907096).

O pegcetacoplan é um peptídeo cíclico peguilado que se liga ao componente C3 do complemento e ao seu fragmento de ativação C3b, impedindo a clivagem de C3 e a ativação subsequente do complemento. Essa inibição proximal bloqueia a ativação das três vias do complemento, prevenindo a formação das convertases de C3 e C5 e reduzindo a inflamação e a lesão tecidual mediadas pelo complemento. O regime padrão consiste na administração de uam dose de 1080 mg por infusão subcutânea duas vezes por semana^38^. Um estudo clínico de fase 3 avaliando o pegcetacoplan em adultos com alto risco de DGF após transplante renal deverá incluir aproximadamente 320 participantes. O desfecho primário é o tempo até a independência de diálise em até 90 dias após o transplante (ClinicalTrials.gov NCT07020832).

### Considerações críticas

O sistema complemento é um componente central da imunidade inata e participa da defesa do hospedeiro, da inflamação e do reparo tecidual. A inibição do complemento no contexto do transplante renal levanta diversas considerações clínicas e mecanísticas.

#### Momento da inibição do complemento

A ativação do complemento pode ocorrer antes do transplante. Morte encefálica e morte circulatória em doadores de órgãos ativam vias do complemento e podem iniciar lesão isquêmica antes da captação do órgão. A maioria dos estudos clínicos administra inibidores do complemento antes da reperfusão do enxerto. Estratégias alternativas, como perfusão *ex vivo* do enxerto com inibidores do complemento ou tratamento do doador antes da captação, requerem investigação adicional.

#### Seleção do alvo no sistema complemento

O componente ou a via ideal do complemento para inibição terapêutica permanecem incertos. Estudos pré-clínicos não estabeleceram qual estratégia de inibição das vias clássica, da lectina ou alternativa oferece a alternativa mais eficaz para prevenir lesão de isquemia–reperfusão, mantendo a segurança. O grau de inibição necessário para eficácia clínica também não está claramente definido. Atividade residual de C5 e ativação persistente a montante podem contribuir para a eficácia limitada observada em alguns estudos com inibição da via terminal.

#### Seleção de pacientes

Ensaios clínicos têm se concentrado em receptores de transplante renal com maior risco de DGF, embora os critérios de inclusão e exclusão variem entre os estudos. A possível interação entre inibição do complemento e outras intervenções que reduzem o risco de DGF, como perfusão hipotérmica ou normotérmica em máquina, ainda não foi totalmente avaliada.

#### Medidas de desfecho

Os desfechos primários variam entre os ensaios clínicos e incluem incidência de DGF, duração da dependência de diálise e função renal entre 90 e 180 dias após o transplante. Essa heterogeneidade limita comparações diretas entre estudos.

#### Segurança

A inibição sistêmica do complemento está associada a maior suscetibilidade a infecções por bactérias encapsuladas. Estratégias de vacinação e profilaxia antibiótica são frequentemente recomendadas durante períodos de inibição do complemento, especialmente em receptores de transplante que recebem imunossupressão concomitante.

#### Considerações regulatórias

O desenvolvimento de múltiplos inibidores do complemento direcionados a diferentes componentes da via, associado ao uso de desfechos clínicos heterogêneos, apresenta desafios para a avaliação regulatória. A relevância clínica dos efeitos do tratamento, particularmente para desfechos de curto prazo como DGF, precisará ser equilibrada com potenciais riscos de segurança.

#### Custo-efetividade

Benefícios econômicos de curto prazo podem decorrer da redução da necessidade de diálise durante episódios de DGF. A custo-efetividade em longo prazo dependerá de melhorias sustentadas na função do enxerto, da redução da falência do enxerto e do perfil global de segurança das estratégias de inibição do complemento.

#### Perspectivas futuras

Considerando os resultados iniciais heterogêneos observados com inibidores do complemento na melhora da recuperação da função renal após o transplante, as incertezas quanto ao momento ideal de administração, ao grau de inibição da via, às medidas de desfecho mais apropriadas e à sua interação com as estratégias atuais e emergentes de perfusão de órgãos sugerem que a indicação clínica ideal, se houver, deverá aguardar os resultados de ensaios clínicos de fase 3.

## PREVENÇÃO DA REJEIÇÃO AGUDA

### Limitações dos regimes imunossupressores baseados em inibidores da calcineurina

O tacrolimo em combinação com o micofenolato, com ou sem corticosteroides, permanece como o regime padrão de imunossupressão de manutenção no transplante renal^39^. A terapia de indução com antagonistas do receptor da interleucina-2 (IL-2R) ou com a globulina antitimócito é frequentemente utilizada com base no risco estimado de rejeição aguda e de função tardia do enxerto^40^. Essas estratégias resultaram em elevadas taxas de sobrevida do paciente e do enxerto no primeiro ano de transplante.

Apesar desses resultados favoráveis de curto prazo, diversas limitações dos regimes baseados em inibidores da calcineurina (CNI) foram identificadas. A elevada variabilidade intraindividual nas concentrações residuais do tacrolimo e o menor tempo dentro da faixa terapêutica estão associados a um maior risco de perda do enxerto, independentemente da adesão ao tratamento^41,42^. Além disso, os ICNs estão associados à nefrotoxicidade e a efeitos adversos sistêmicos metabólicos e cardiovasculares que podem comprometer os desfechos de longo prazo dos receptores de transplante renal^43^.

A globulina antitimócito recebeu recentemente aprovação regulatória para profilaxia da rejeição aguda em receptores de transplante renal^44^. Entretanto, seu uso está associado a um aumento dose-dependente de eventos adversos, incluindo infecções bacterianas e virais^45^.

Agentes biológicos de longa duração podem proporcionar efeitos farmacodinâmicos mais estáveis e regimes posológicos simplificados. Essas características podem melhorar a adesão ao tratamento e potencialmente contribuir para melhores desfechos do enxerto em longo prazo^46^.

### Terapia anti-CD2

O siplizumabe é um anticorpo monoclonal humanizado direcionado ao receptor CD2 que tem sido investigado em terapias de indução e em protocolos de tolerância. Seu mecanismo de ação inclui depleção seletiva de células T de memória efetoras, redução da ativação e proliferação de células T e preservação relativa ou enriquecimento de células T reguladoras (Tregs), o que pode promover tolerância imunológica e reduzir respostas aloimunes^47,48^.

Ensaios clínicos de fase 2 avaliando segurança, tolerabilidade, farmacocinética e farmacodinâmica de doses crescentes de siplizumabe em comparação com globulina antitimócito de coelho em receptores de transplante renal *de novo* foram concluídos (NCT06365437; NCT04311632). Estudos de fase 3 são esperados.

### Bloqueio de vias de coestimulação

Respostas aloimunes eficazes requerem sinais de coestimulação gerados por interações entre células apresentadoras de antígeno e linfócitos T^49^. As vias coestimuladoras envolvem receptores da superfamília das imunoglobulinas (CD80, CD86, CD28, CTLA-4, ICOS, ICOSL, PD-1 e PD-L1) e da superfamília do fator de necrose tumoral (CD40 e CD154)^50^.

Agentes biológicos direcionados a essas vias interferem seletivamente em receptores imunes de membrana e podem reduzir efeitos *off-target* não relacionados ao sistema imune quando comparados com estratégias imunossupressoras menos seletivas.

### Via CD40–CD40L

CD40 e CD40L (CD154) são expressos em múltiplos tipos celulares. O CD40 é expresso constitutivamente em linfócitos B e células mieloides, enquanto o CD40L é expresso princi­palmente em linfócitos T ativados e plaquetas. A interação entre CD40 e CD40L ativa vias de sinalização que regulam respostas imunes, inflamação, hematopoiese, trombose e biologia tumoral^51^.

A inibição da sinalização CD40–CD40L interfere tanto nas respostas aloimunes celulares quanto nas humorais. Essa via é necessária para a ativação completa de células T e para a ativação e diferenciação de células B, incluindo a geração de anticorpos específicos contra o doador. O bloqueio dessa interação por anticorpos monoclonais direcionados contra CD154 ou CD40 prolonga a sobrevida do aloenxerto e reduz a rejeição em modelos pré-clínicos de transplante^52^.

Anticorpos anti-CD154 exercem diversos efeitos imunológicos, incluindo:

- inibição da sinalização CD40–CD154;
- redução da expansão clonal de células B e da produção de anticorpos;
- modulação da ativação de células imunes inatas;
- promoção de células T reguladoras induzíveis^52^.

Estudos pré-clínicos sugerem que o bloqueio de CD154 pode ser mais eficaz do que o bloqueio de CD40 na prevenção da rejeição. Uma meta-análise em modelos de transplante em primatas não humanos relatou indução de tolerância aproximadamente três vezes maior com terapia anti-CD154 em comparação com agentes anti-CD40^50^.

O desenvolvimento clínico de dois anticorpos anti-CD40, iscalimabe e ASKP1240, foi interrompido devido à falta de benefício clínico suficiente.

### Terapias anti-CD154

Os primeiros anticorpos anti-CD154 foram associados ao aumento do risco de eventos tromboembólicos. Essa complicação provavelmente resulta da expressão de CD154 em plaquetas ativadas e da interação entre a região Fc do anticorpo e os receptores FcγRIIa plaquetários, levando à ativação e à agregação plaquetária.

Agentes mais recentes com domínios Fc modificados ou com fragmentos Fab isolados foram desenvolvidos, reduzindo a ativação plaquetária e o risco tromboembólico^52^.

- Tegoprubart é um anticorpo IgG1 humanizado de segunda geração direcionado ao CD154 com região Fc modificada para minimizar a interação com FcγRIIa. Está sendo avaliado em ensaios clínicos de fase 2 para prevenção da rejeição aguda após o transplante renal (NCT05983770).
- Frexalimabe é outro anticorpo monoclonal humanizado anti-CD154 de segunda geração em investigação para doenças autoimunes e para o transplante^53^.
- TNX-1500 é um anticorpo anti-CD154 de terceira geração, atualmente em desenvolvimento clínico inicial para transplante renal (NCT07204080).

### Via CD80/CD86–CD28–CTLA-4

CD28 e a proteína CTLA-4 (*cytotoxic T-lymphocyte–associated protein* 4) regulam a ativação de células T por meio de interações com CD80 e CD86 expressos em células apresentadoras de antígeno.

- CD28 é expresso constitutivamente em células T *naïve* e fornece um sinal coestimulador essencial para ativação, proliferação, produção de citocinas e sobrevivência das células T após o reconhecimento do antígeno^54^.
- CTLA-4 é regulado positivamente após a ativação das células T e se liga a CD80/CD86 com maior afinidade do que CD28, inibindo a ativação e proliferação das células T por mecanismos intrínsecos e extrínsecos, incluindo trans-endocitose de CD80/CD86 e modulação da função de células T reguladoras^55^.

O belatacepte, uma proteína de fusão composta pelo domínio extracelular de CTLA-4 ligado ao fragmento Fc de IgG1, bloqueia seletivamente a coestimulação mediada por CD28. Embora aprovado para transplante renal, seu uso mais amplo tem sido limitado pela maior incidência de rejeição aguda em comparação com regimes baseados em CNI^56^.

### Terapia anti-CD28

O bloqueio seletivo de CD28 pode fornecer imunomodulação, preservando simultaneamente a sinalização inibitória mediada por CTLA-4. Como o bloqueio de CD28 não interfere na sinalização coinibitória mediada por CTLA-4 nem nas interações PD-L1–CD80, a função das células T reguladoras pode ser preservada, melhorando potencialmente o controle das respostas aloimunes^57,58,59^.

O pegrizeprumente (FR104) é um fragmento Fab′ peguilado monovalente humanizado que antagoniza a sinalização de CD28. Ao bloquear seletivamente a ativação de células T mediada por CD28 enquanto preserva as vias CTLA-4 e PD-L1, pode-se manter mecanismos regulatórios do sistema imune^60^.

Estudos clínicos iniciais avaliaram doses intravenosas variando de 0,02 mg/kg a 1,5 mg/kg, demonstrando ocupação do receptor e efeitos farmacodinâmicos dependente da dose administrada^61^.

Um estudo clínico de fase 1/2 (NCT04837092) está avaliando segurança, tolerabilidade, farmacocinética e eficácia preliminar do pegrizeprumente em receptores de transplante renal com rins de doadores de critérios padrão. Um ensaio de fase 2 (NCT07290777) comparará pegrizeprumente com tacrolimo na prevenção de rejeição aguda após o transplante renal.

### Avaliação crítica

Diversos desafios permanecem no desenvolvimento clínico e na implementação de agentes biológicos que bloqueiam vias de coestimulação em receptores de transplante renal.

#### Limitações das estratégias utilizando bloqueadores de coestimulação

Apesar de um forte racional mecanístico, o desenvolvimento clínico e a adoção desses agentes têm sido limitados. Os fatores incluem elevadas taxas de rejeição aguda, complicações infecciosas (especialmente infecções virais), risco de doença linfoproliferativa pós-transplante (PTLD), incertezas quanto à segurança em longo prazo e eficácia comparativa limitada em relação aos regimes baseados em CNI^49^.

#### Eficácia e segurança dos anticorpos anti-CD154

A maior eficácia de terapias anti-CD154 em comparação com anti-CD40 observada em modelos de primatas não humanos precisa ser confirmada em estudos clínicos. O monitoramento contínuo de eventos tromboembólicos permanece necessário, apesar das modificações na região Fc em anticorpos mais recentes.

#### Eficácia e segurança dos anticorpos anti-CD28

A eficácia clínica do bloqueio seletivo de CD28 ainda requer validação adicional. A maior incidência de rejeição aguda observada com belatacepte tem sido associada à presença de células T CD28-negativas circulantes, que podem não ser afetadas por antagonistas de CD28^62^. Em pacientes com rejeição resistente ao belatacepte, a adição de um inibidor de mTOR foi associada à resolução da rejeição e à redução das células T de memória efetora CD8^+^CD28^−^ circulantes^63^.

#### Monitoramento terapêutico

É necessário um melhor entendimento das propriedades farmacocinéticas e farmacodinâmicas dos agentes biológicos. Avaliações além das concentrações plasmáticas e da ocupação de receptores periféricos podem ser necessárias para otimizar estratégias terapêuticas^56^.

#### Segurança dos bloqueadores de coestimulação

Considerações de segurança permanecem críticas, incluindo o risco de PTLD — particularmente em receptores soronegativos para o vírus *Epstein–Barr* tratados com belatacepte — e o risco de tuberculose em regiões com alta prevalência endêmica.

#### Desafios para demonstrar superioridade clínica

O desenho de ensaios clínicos apresenta desafios. Demonstrar superioridade utilizando desfechos compostos regulatórios atuais (rejeição aguda, perda do enxerto, óbito ou perda de seguimento) é difícil devido às baixas taxas de eventos nos grupos controle contemporâneos. Diferenças na função renal aos 12 meses também podem exigir grandes populações de estudo para detectar efeitos significativos.

Embora a relevância clínica desse desfecho composto seja debatida^64^, a identificação e a validação de marcadores substitutos para sobrevida do enxerto em longo prazo continuam desafiadoras. O sistema de pontuação *iBox* integra parâmetros clínicos, funcionais, imunológicos e histológicos, incluindo tempo desde o transplante, taxa de filtração glomerular estimada, proteinúria, achados de biópsia e anticorpos específicos contra o doador^65^.

A pontuação *iBox* medida um ano após o transplante demonstrou desempenho prognóstico superior para perda do enxerto em comparação com rejeição aguda comprovada por biópsia ocorrida no primeiro ano pós-transplante^66^. O sistema *iBox* foi qualificado pela Agência Europeia de Medicamentos (EMA) em 2022 como desfecho secundário de eficácia para ensaios clínicos em transplante renal. Sua qualificação como desfecho substituto para decisões regulatórias está atualmente em revisão pela *Food and Drug Administration* (FDA) dos Estados Unidos.

O uso do escore *iBox* em 12 meses como desfecho co-primário, comomarcador prognóstico para a sobrevida do enxerto censurada para morte em longo prazo, está em investigação.

#### Regime de imunossupressão ideal

O regime imunossupressor de base ideal para o bloqueio de coestimulação permanece incerto. Taxas de rejeição comparáveis à terapia padrão foram relatadas principalmente com belatacepte combinado a inibidores de mTOR^67,68^.

Estudos pré-clínicos e translacionais sugerem que a combinação de bloqueio de coestimulação e inibição de mTOR pode promover tolerância específica ao doador, sobrevida prolongada do enxerto e quimerismo misto estável^69,70,71^.

Entretanto, o perfil de tolerabilidade dos inibidores de mTOR e considerações regulatórias limitaram o avanço adicional dessa estratégia. Além disso, análises de custo-efetividade serão necessárias para determinar se potenciais melhorias na função e na sobrevida do enxerto compensam os custos associados às terapias biológicas e ao monitoramento de longo prazo.

## TRATAMENTO DA REJEIÇÃO MEDIADA POR ANTICORPOS

A rejeição mediada por anticorpos (*antibody-mediated rejection* – AMR) é caracterizada pela ligação de anticorpos específicos contra o doador (DSA) a antígenos endoteliais do enxerto, ativação do complemento, lesão endotelial e recrutamento de células efetoras inflamatórias, incluindo células *natural killer* (NK). Esses processos resultam em inflamação microvascular e lesão progressiva do aloenxerto^72^.

Uma revisão sistemática recente relatou incidência de AMR aguda variando de 1,1% a 21,5%, enquanto a AMR crônica ocorre em aproximadamente 7,5%–20,1% dos receptores dentro de 10 anos após o transplante. A AMR é um fator de risco independente para falência do enxerto censurada para morte^73^.

As recomendações atuais de tratamento baseiam-se amplamente em consenso de especialistas. O grupo de trabalho da Sociedade Internacional de Transplantes recomenda plasmaférese combinada com imunoglobulina intravenosa (IVIG) para AMR precoce, juntamente com otimização da imunossupressão de base. Para AMR tardia ativa ou crônica ativa, recomenda-se geralmente a otimização da imunossupressão de manutenção^74^.

Diversos estudos clínicos de fase II e III estão avaliando estratégias terapêuticas direcionadas a diferentes componentes da resposta imune humoral, incluindo:

- inibição da sinalização da interleucina-6 (IL-6);
- depleção de células B e plasmócitos;
- clivagem enzimática de DSAs circulantes;
- inibição seletiva do complemento;
- terapias direcionadas ao CD38^75,76^.

### Terapias anti-CD38

O CD38 é uma glicoproteína transmembrana multifuncional expressa em ampla variedade de células hematopoiéticas e não hematopoiéticas. Sua expressão aumenta após a ativação celular, sendo observada em células T, células B e células NK, onde contribui para a regulação de diferenciação celular, proliferação, secreção de citocinas, apoptose, fagocitose, quimiotaxia e atividade citotóxica de células NK^77^.

O CD38 também regula o metabolismo intracelular da nicotinamida adenina dinucleotídeo (NAD^+^) e influencia a função mitocondrial e metabólica^78^.

O bloqueio do CD38 leva à depleção de plasmócitos, plasmablastos, células B e células NK. Os mecanismos envolvidos incluem:

- citotoxicidade celular dependente de anticorpos (ADCC);
- citotoxicidade dependente de complemento (CDC);
- fagocitose celular dependente de anticorpos (ADCP);
- inibição da atividade enzimática do CD38.

A contribuição relativa desses mecanismos depende do epítopo de ligação e da estrutura do anticorpo monoclonal utilizado^79^.

O felzartamabe, o daratumumabe e o isatuximabe são anticorpos monoclonais anti-CD38 que têm sido utilizados *off-label* no tratamento da AMR^75^.

Um estudo recente de fase 2, duplo-cego, controlado por placebo, relatou resolução da atividade histológica de AMR em 82% dos pacientes tratados com felzartamabe na semana 24, em comparação com 20% no grupo placebo. O tratamento foi associado a:

- redução da atividade histológica de AMR;
- diminuição dos escores de inflamação microvascular;
- redução de transcritos induzíveis por interferon-γ;
- diminuição de assinaturas gênicas de células NK;
- redução de DNA livre de células derivado do doador.

Não foram observadas alterações significativas nos níveis de DSA, e apenas efeitos mínimos foram detectados nos transcritos associados à lesão endotelial. A recorrência molecular da rejeição foi observada aproximadamente seis meses após a interrupção do tratamento^80^.

Um ensaio clínico multicêntrico de fase 3 avaliando felzartamabe em receptores de transplante renal com AMR tardia está atualmente recrutando participantes (NCT06685757).

### Inibição do complemento e estratégias anti-C3

A ativação do sistema complemento é um componente central da patogênese da AMR. A ligação de DSAs, geralmente da classe IgG, a antígenos endoteliais ativa a via clássica do complemento, gerando fragmentos como C4d e C3d. Esses fragmentos ligam-se covalentemente às superfícies endoteliais e servem como marcadores diagnósticos de ativação do complemento na AMR.

A formação do complexo de ataque à membrana (C5b-9) pode causar lesão direta das células endoteliais. Entretanto, dados recentes sugerem que a ativação terminal do complemento pode ser menos proeminente na AMR tardia ou crônica, enquanto fragmentos mais proximais, como C4d e C3d, são detectados de forma mais consistente e podem ter maior relevância clínica^81^.

As evidências que sustentam o uso do inibidor de C5 eculizumabe para tratamento de AMR permanecem limitadas, e seu uso rotineiro não é recomendado atualmente. Estratégias alternativas direcionadas a componentes proximais do complemento, incluindo inibidores da C1 esterase e anticorpos monoclonais como sutimlimabe, estão em investigação.

Uma abordagem adicional para inibição do complemento envolve terapias baseadas em RNA de interferência (*RNA interference* – RNAi). Os pequenos RNAs de interferência (siRNA) promovem silenciamento gênico pós-transcricional ao induzir degradação do RNA mensageiro (mRNA) alvo.

Essas moléculas de RNA de dupla fita, geralmente com 21–23 nucleotídeos, são incorporadas ao complexo de silenciamento induzido por RNA (RISC), no qual a fita anti­senso direciona a clivagem específica do mRNA comple­mentar, impedindo sua tradução e reduzindo a expressão da proteína^82^.

Diversas terapias baseadas em siRNA já foram aprovadas para uso clínico, incluindo tratamentos para:

- amiloidose hereditária por transtirretina (patisiran);
- porfiria hepática aguda (givosiran);
- hiperoxalúria primário tipo 1 (lumasiran);
- hipercolesterolemia familiar (inclisiran).

O ALXN203 é um siRNA de dupla fita não codificante conjugado à N-acetilgalactosamina (GalNAc), permitindo o seu direcionamento direto aos hepatócitos por meio do receptor de asialoglicoproteína. A molécula reduz a produção hepática do componente C3 do complemento por degradação do mRNA de C3.

Um ensaio clínico multicêntrico de fase 2 está recrutando receptores de transplante renal com AMR para avaliar a eficácia e segurança do ALXN203 (NCT06744647).

### Avaliação crítica

Diversos fatores complicam o desenvolvimento terapêutico para AMR.

#### Heterogeneidade da AMR

A AMR é uma condição relativamente rara, complexa e heterogênea, com critérios diagnósticos em constante evolução, os quais incorporam características clínicas, histológicas e moleculares. Inflamação microvascular com ou sem DSAs detectáveis está associada a desfechos adversos do enxerto^83^.

#### Atividade da AMR

Análises histológicas e moleculares demonstram considerável variabilidade na atividade da doença, mesmo quando critérios diagnósticos rigorosos são aplicados. Essa variabilidade pode refletir fenótipos distintos ou diferentes estágios da progressão da doença^84,85^.

#### Desafios dos estudos clínicos

essa heterogeneidade dificulta o desenho de ensaios clínicos e a avaliação regulatória de novas terapias.

#### Tratamento contínuo

Dados emergentes sugerem que terapia sustentada pode ser necessária para manter o controle da doença, uma vez que a recorrência da rejeição foi observada após interrupção do tratamento em estudos clínicos iniciais^80^.

#### Segurança em longo prazo

O perfil de segurança em longo prazo dessas terapias sob admini­stração crônica permanece incerto. Estratégias de monitoramento, programas de vacinação, profilaxia antimicrobiana e vigilância para infecções endêmicas serão necessários para avaliação do risco-benefício em longo prazo e possíveis impactos na qualidade de vida.

## Considerações Finais

O desenvolvimento de imunoterapias direcionadas representa uma área importante de investigação no transplante renal. As estratégias terapêuticas atualmente em estudo abordam diversos desafios clínicos relevantes, incluindo o manejo de pacientes altamente sensibilizados, terapias de depleção de linfócitos seletivas que promovem um ambiente imunológico tolerogênico após a reconstituição homeostática, mitigação da lesão de isquemia–reperfusão, prevenção de rejeição aguda sem toxicidade associada a inibidores da calcineurina e tratamento da rejeição mediada por anticorpos (Figura 1).

**Figura 1:** Posicionamento terapêutico proposto de agentes emergentes no transplante renal.

Estratégias terapêuticas emergentes buscam proporcionar benefícios incrementais em relação ao regime atual de tratamento padrão, que consiste em indução com globulina antitimócito de coelho (rATG) e imunossupressão de manutenção com tacrolimo, micofenolato de mofetila e corticosteroides. Diversas abordagens estão em investigação. Primeiro, a dessensibilização com imlifidase pode ampliar o acesso ao transplante para candidatos altamente sensibilizados. Segundo, a inibição da cascata do complemento pode reduzir a incidência e a duração da função retardada do enxerto e favorecer uma recuperação mais completa da função do aloenxerto. Terceiro, a terapia de indução seletiva com siplizumabe pode proporcionar imunossupressão eficaz ao mesmo tempo em que reduz os eventos adversos associados à rATG. Quarto, a imunossupressão de manutenção com novos bloqueadores da via de coestimulação pode eliminar as toxicidades metabólicas, cardiovasculares e nefrotóxicas associadas aos inibidores da calcineurina, ao mesmo tempo em que mantém a função renal estável. Além disso, o bloqueio da coestimulação tem sido associado à maior sobrevivência de células T reguladoras (Treg), à redução da proliferação de células T efetoras, à diminuição das respostas de células B de memória e a menores níveis de anticorpos específicos contra o doador. Por fim, novas abordagens terapêuticas para o tratamento da rejeição mediada por anticorpos tardia, tanto aguda quanto crônica ativa, podem retardar a progressão da disfunção do aloenxerto renal. Esses potenciais benefícios devem ser comparados com os riscos associados à intensificação ou introdução de novas formas de imunossupressão, incluindo infecções bacterianas, virais e fúngicas, bem como neoplasias associadas a vírus oncogênicos, como a doença linfoproliferativa pós-transplante. Assim, análises rigorosas de risco-benefício e de custo-efetividade serão essenciais para determinar o valor clínico dessas estratégias imunossupressoras emergentes, com o objetivo final de reduzir a perda do enxerto, o retorno à diálise, a mortalidade e melhorar a qualidade de vida dos receptores de transplante renal.

Os desafios na tradução da inibição de mecanismos promissores, observados em estudos pré-clínicos, em benefícios clínicos são bem conhecidos na área de transplantes. A interrupção precoce do desenvolvimento do fingolimode^86^, do inibidor de JAK3 tofacitinibe^87^ e do inibidor de tirosina quinase sotrastaurina^88^ são exemplos representativos.

O papel clínico futuro dessas terapias permanece incerto, e sua integração aos regimes imunossupressores padrão dependerá da demonstração de eficácia, segurança e custo-efetividade. Estratégias combinadas direcionadas a vias imunológicas complementares podem ser necessárias para alcançar o controle ideal das respostas aloimunes.

Considerações de segurança incluem a maior suscetibilidade a infecções bacterianas associada à inibição do complemento, maior risco de infecções virais associado ao bloqueio de coestimulação, possível reativação viral e complicações virais oncogênicas.

Dados de segurança em longo prazo estão disponíveis para o inibidor de C5 eculizumabe. A análise de 46.316 eventos adversos relatados no *FDA Adverse Event Reporting System* identificou 461 termos preferenciais consistentes com a bula do medicamento, incluindo fadiga, nasofaringite, infecção meningocócica, febre e anemia^89^.

O bloqueio de coestimulação está associado à preservação de células T reguladoras, redução da proliferação de células T efetoras, diminuição de respostas de células B de memória e menores níveis de anticorpos específicos contra o doador.

Como essas terapias inibem principalmente a ativação de células T *naïve* enquanto preservam respostas de memória já estabelecidas, a incidência de infecções oportunistas pode depender da exposição prévia a patógenos e da imunossupressão concomitante^90^.

Estratégias de monitoramento farmacodinâmico para terapias biológicas direcionadas ainda são limitadas. As avaliações atuais restringem-se principalmente à medição de componentes circulantes do complemento ou à ocupação de receptores, o que pode não refletir completamente os efeitos imunológicos subsequentes.

A meia-vida prolongada e a possibilidade de administração subcutânea de múltiplos agentes biológicos emergentes podem permitir intervalos de dose mais longos e melhor adesão ao tratamento. Investigações adicionais serão neces­sárias para determinar combinações terapêuticas ideais, estratégias de uso sequencial e perfis de segurança em longo prazo.

## Data Availability

Este estudo não gerou ou analisou novos dados.
